# Benefits of Targeted Molecular Therapy to Immune Infiltration and Immune-Related Genes Predicting Signature in Breast Cancer

**DOI:** 10.3389/fonc.2022.824166

**Published:** 2022-03-04

**Authors:** Fahai Chen, Jianmin Fang

**Affiliations:** ^1^ CEO Office, RemeGen Co. Ltd., Yantai, China; ^2^ School of Life Science and Technology, Tongji University, Shanghai, China

**Keywords:** breast cancer, tumor microenvironment, immune infiltration, ESTIMATE algorithm, CIBERSORT algorithm

## Abstract

**Background:**

This study aimed to investigate the tumor-related infiltrating lymphocytes (TILs) affecting the response of trastuzumab and identify potential biomarkers based on immune-related genes to improve prognosis and clinical outcomes of targeted therapies in breast cancer.

**Methods:**

Estimation of stromal and immune cells in malignant tumors using expression data (ESTIMATE) was adopted to infer the fraction of stromal and immune cells through utilizing gene expression signatures in breast tumor samples. Cell-type identification by estimating relative subsets of RNA transcript (CIBERSORT) algorithm was applied to characterize cell composition of 22 lymphocytes from breast cancer tissues using their gene expression profiles. Immune-related genes were collected from the Immunology Database and Analysis (ImmPort). Univariate and multivariate Cox regression analyses were performed to identify the significant independent risk factors associated with poor overall survival (OS) and breast cancer-specific survival (BCSS) of breast cancer patients. Hub genes were identified based on the protein–protein interaction (PPI) network analysis.

**Results:**

Based on the ESTIMATE algorithm, a significant reduction of stromal scores was observed in tumor tissues and pretreated tumor tissues compared with nontumor and posttreated tumor tissues, respectively, while immune scores failed to present notably statistical differences between both groups. However, from the results of the univariate Cox regression analysis, the immune score was identified to be remarkably associated with the poor OS for breast cancer patients. Subsequently, the infiltrating lymphocytes were evaluated in tumor tissues based on the CIBERSORT algorithm. Furthermore, significance analysis identified 1,244 differentially expressed genes (DEGs) from the GSE114082 dataset, and then 91 overlapping immune-related DEGs were screened between GSE114082 and ImmPort datasets. Subsequently, 10 top hub genes were identified and five (IGF1, ADIPOQ, PPARG, LEP, and NR3C1) significantly correlated with worse OS and BCSS on response to trastuzumab in breast cancer patients.

**Conclusions:**

This study provided an insight into the immune score based on the tumor-related infiltrating lymphocytes in breast cancer tissues and demonstrates the benefits of immune infiltration on the treatment of trastuzumab. Meanwhile, the study established a novel five immune-related gene signature to predict the OS and BCSS of breast cancer treated by trastuzumab.

## Introduction

The tumor microenvironment (TME) is composed of various cell types (such as stromal cells, tumor cells, and immune cells) and extracellular components (such as cytokines, growth factors, hormones, etc.) that are involved in each progression stage of oncogenesis (1). The TME does not only contribute to tumor initiation, progression, and metastasis but also has a powerful effect on therapeutic responses. Moreover, the multitude of interactions between tumor cells and the TME help tumor cells evade immunological surveillance and devote themselves to environment-mediated drug resistance (1, 2). Tumor-infiltrating lymphocytes (TILs) comprised an extensive part of tumor-infiltrating immune cells and were considered to inhibit tumor growth resulting in the improved clinical outcomes of immuno- and chemotherapy in melanoma, colorectal cancer, and ovarian cancer (3–5). Breast cancer was generally thought to have a weak immunogenicity, but recent studies have demonstrated high levels of TILs occurring in HER2^+^ and basal-like subtypes which are associated with good prognosis and with response to certain therapies (6, 7). Therefore, the immune system contributes a promising option, with targeted immunotherapeutic approaches to selectively regulate the body’s immune system to eradicate tumors showing a potent promise in clinical studies (8, 9).

The advent of targeted therapeutics has intensively changed the administration landscape across a variety of malignancies, including breast cancer. Immune checkpoint antagonists targeting PD-1, PD-L1, and CTLA-4 have reformed cancer therapy and demonstrated the power of treating the immune system benefiting multiple cancer types (10, 11). Although targeted therapeutics is regarded as a potent hope for various cancers, a considerable number of patients failed to benefit from the assignment or acquired drug resistance (12). Trastuzumab, a monoclonal antibody targeted against HER2, has remarkably improved clinical outcomes for HER2-positive breast cancer patients (13). However, despite significant advances, only partial metastatic patients responded to trastuzumab and approximately 60% suffered resistance after initial response (14). The comprehensive understanding of tumor-associated stromal cells and immune cells in breast tumor tissues may provide important insights into tumor biology and its therapeutic response, and then explores the potential strategies to achieve significant clinical benefits of targeted treatment by regulating factors of the TME.

In the present study, the stromal scores and tumor-related lymphocytes involved in the response of targeted molecular therapy were characterized *via* the ESTIMATE algorithm and Cibersort algorithm. Subsequently, the diagnostic value of hub genes of immune-related genes associated with outcomes of trastuzumab treatment was evaluated based on biological function and signaling pathway enriched analysis of signaling pathways in breast cancer. These findings could provide a novel insight into the response of tumor-related lymphocytes and immune-related genes to targeted molecular therapy and trastuzumab treatment.

## Methods

### Data Collection and Processing

The Cancer Genome Atlas-BReast invasive CArcinoma (TCGA-BRCA) gene expression level 3 data were downloaded from TCGA data portal (https://portal.gdc.cancer.gov/repository/), and the clinical information of breast cancer samples was collected from the UCSC Xena (https://xena.ucsc.edu/public). Of them, 467 patients treated by targeted molecular therapy and 99 nontumor samples were screened from TCGA-BRCA database. GSE114082 dataset was collected from the GEO database (https://www.ncbi.nlm.nih.gov/geo/). GSE114082 dataset contains the gene expression profile from frozen core biopsies of 17 breast cancer patients before and after brief-exposure treatment with single-agent neoadjuvant trastuzumab. The platform was based on GPL14951 (Illumina HumanHT-12 WG-DASL V4.0 R2 expression bead chip). The GSE114082 dataset was evaluated by consistency check (**Supplementary Figure 1**).

### ESTIMATE Algorithm

Estimation of stromal and immune cells in malignant tumor tissues using expression data (ESTIMATE) utilizes the advantage of the unique properties of the cancer samples to infer tumor cellularity as well as the infiltration of normal cells. By conducting single-sample gene set-enrichment analysis (ssGSEA), stromal and immune scores were calculated to predict the level of infiltrating stromal and immune cells, and these form the fundamental ESTIMATE algorithm (15). The matrix data of gene expression amounts were normalized with the limma package of the R software, and then stromal and immune scores were calculated by applying the ESTIMATE algorithm.

### CIBERSORT Estimation

To quantify the proportions of 22 TILs in breast cancer tissues, we applied CIBERSORT to determine the abundance of cell types in a mixed cell population using normalized data. CIBERSORT is a method for characterizing the cell composition of complex tissues based on gene expression profiles. According to an input matrix of reference gene expression signatures, the CIBERSORT collectively estimates the relative proportions of each immune-cell type (16). The gene expression data were uploaded to the CIBERSORT web portal (https://cibersort.stanford.edu/), and then the algorithm was conducted based on the LM22 signature and 1,000 permutations. The visualization of results was performed using the R programming language.

### Identification of DEGs

For GEO data, the R programming language (the limma package) was utilized to screen differentially expressed genes (DEGs) between the untreatment breast group and trastuzumab treatment breast group. The screen criteria were an adjusted *p* value of <0.05 and an absolute log2 fold change (log2FC) of >1.5 or <−1.5.

### Immune-Related Gene Extraction

Immune-related gene (IRGs) data were obtained from the ImmPort database, and overlapping immune-related genes were screened from the GSE114082 dataset for further analysis.

### Functional and Pathway Enrichment Analysis

Gene Ontology (GO) and Kyoto Encyclopedia of Genes and Genomes (KEGG) pathway enrichment analyses were conducted usimg R programming language to analyze the potential biological processes (BP), cellular components (CC), molecular functions (MF), and pathway of the overlapping immune-related DEGs. The R programming language was applied to visualize the results of GO analysis and KEGG pathway enrichment analysis. *p* < 0.05 was set as the threshold value.

### PPI Network

The online tool, search tool for the retrieval of interacting genes (STRING, https://string-db.org/), was performed to assess the interactive relationships of the overlapping DEGs. The STRING interactome was selected as the PPI database that is with medium (400) to high (1,000) confidence score. The Cytoscape software (version 3.8.2) was then used to construct and visualize the PPI network of common immune-related DEGs. The plugin cytoHubba was applied to screen the top 10 hub genes from the PPI network.

### ROC Curves and Survival Analysis

The ROC curve, a comprehensive index conducted to assess the sensitivity and specificity of continuous variables, was applied to evaluate the risk score and diagnostic capability of the top 10 hub genes as biomarkers in distinguishing breast cancer from normal controls, respectively. A *p* value of <0.05 was considered statistically significant. The Kaplan–Meier plotter (KM plotter) was performed using R programming language to predict the prognostic value of the hub genes in breast cancer patients. The patients were stratified into two groups according to the hug gene expression levels (high vs. low expression) and then analyzed the overall survival (OS), breast cancer-specific survival (BCSS), and distant metastasis-free survival (DMFS) based on these categories. The hazard ratios (HRs), 95% confidence intervals, and log-rank *p*-values were shown.

### Statistical Analysis

All statistical analyses were performed using R software (version 4.0.5) and GraphPad Prism v6.00 (GraphPad Software Inc., USA). The *t*-test was used to assess paired samples or a nonparametric Wilcoxon rank-sum test for unpaired samples as appropriate. One-way ANOVA was employed for multiple groups of normalized data. The univariate and multivariate Cox regression analyses were conducted to assess the remarkable prognostic factors. The time-dependent receiver operating characteristic (ROC) curve was applied to evaluate the predictive accuracy of the diagnostic signatures of hub genes. The Kaplan–Meier curve log-rank test was applied to assess the statistical significance of the survival rates between different risk hub genes. *p* < 0.05 was considered statistically significant.

## Results

### Estimation of Infiltrating Stromal and Immune Cells

**Figure 1:**
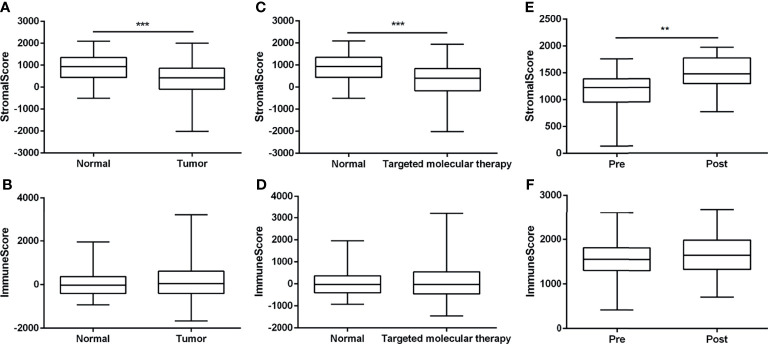
The assessment of stromal scores and immune scores using the ESTIMATE algorithm. **(A, B)** The stromal and immune scores between nontumor tissues and breast tumor tissues based on the TCGA-BRCA dataset. **(C, D)** The stromal and immune scores of 99 normal samples and 467 tumor samples with the treatment of targeted molecular therapy, based on TCGA-BRCA dataset. **(E, F)** The stromal scores and immune scores between breast tumor tissues whether treated by trastuzumab or not, based on the GSE114082. The data were presented as the mean ± SD, normal vs. tumor, pretreatment vs. posttreatment. ^**^ *p* < 0.01; ^***^ *p* < 0.001.

To explore the proportion of infiltrating cells in tumor tissues, we utilized the ESTIMATE algorithm to assess two gene signatures: a stromal signature that represents the presence of stroma in tumor tissue and an immune signature that represents the attendance of immune cells in tumor tissue. According to the TCGA-BRCA datasets, stromal scores in normal tissues showed higher levels compared with the tumor tissues (**Figure 1A**). Compared with normal tissues, a higher immune score was determined in tumor tissues, but there were no significant statistical differences (**Figure 1B**). However, the administration of targeted molecular therapy significantly decreased the stromal score in comparison with normal samples, but there was no statistical significance on the immune score between groups with targeted therapy or not (**Figures 1C, D** **)**. Furthermore, we assessed the stromal scores and immune scores based on the GSE114082 datasets, and a significant increase in stromal signature scores was observed after treatment of trastuzumab, but there were no significant statistical differences on the immune scores in the pretreatment samples compared with the posttreatment samples (**Figures 1E, F** **)**.

Moreover, the Cox regression analysis was performed to evaluate the independent indicators of OS in breast cancer. In the univariate model, age, tumor–node–metastasis (TNM) stage, and immune score were significantly related to OS in breast cancer (**Table 1**). The following multivariate analysis identified that age and TNM stage were the independent indicators of unfavorable OS in breast cancer. However, the stromal score and the immune score failed to attain a significant relation to OS in multivariate Cox regression analysis (**Table 1**). In samples with targeted molecular therapy, stromal score and immune score showed unmet relation to OS in both univariate and multivariate Cox regression analyses (**Supplementary Table 1**). Because of the lack of key clinical data in the GEO datasets, we failed to assess the independent risk indicators of OS in breast cancer treated or not treated by trastuzumab. The results indicated that the negative immune score as an independent risk factor was consistently associated with unfavorable OS.

**Table 1:** Cox proportional hazards regression model analysis of overall survival based on TCGA-BRCA database.

Variables	Univariate analysis		Multivariate analysis	
	HR (95% CI)	*p*	HR (95% CI)	*p*
Age (≥65 vs. <65)	2.17 (1.56, 3.03)	**<0.001**	2.43 (1.73, 3.41)	**<0.001**
TNM stage (II vs. I)	1.71 (0.98, 3)	0.061	1.89 (1.08, 3.32)	**0.026**
TNM stage (III vs. I)	3.17 (1.76, 5.71)	**<0.001**	3.63 (2.01, 6.55)	**<0.001**
TNM stage (IV vs. I)	13.48 (6.57, 27.66)	**<0.001**	14.62 (7.08, 30.18)	**<0.001**
Stromal score (positive vs. negative)	1.08 (0.76, 1.53)	0.687	–	–
Immune score (positive vs. negative)	0.63 (0.45, 0.87)	**0.006**	0.74 (0.52, 1.03)	0.077

Statistically significant p-values are given in bold, p < 0.05.

HR, hazards ratio; CI, confidence interval.

### Estimation of Immune Cell-Type Fractions

**Figure 2:**
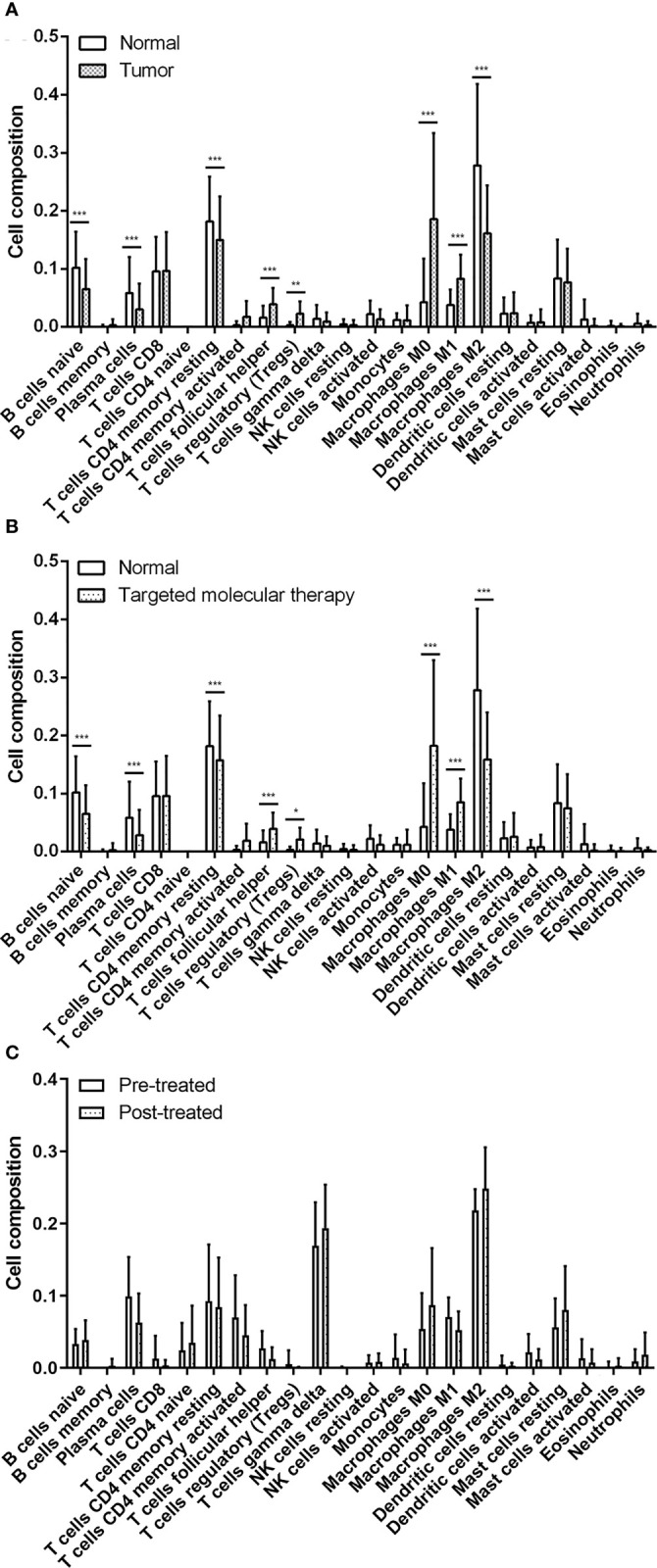
The evaluation of tumor-related infiltrating lymphocytes using the CIBERSORT algorithm. **(A)** Significant statistical analysis of tumor-related infiltration lymphocytes between normal samples and breast tumor samples. **(B)** Significant statistical analysis of tumor-related infiltration lymphocytes between normal tissues and breast tumor samples with targeted molecular therapy. **(C)** Significant statistical analysis of tumor-related infiltration lymphocytes between breast tumor samples before and after treated by trastuzumab. The data were presented as the mean ± SD, normal vs. tumor, pretreatment vs. posttreatment. ^*^ *p* < 0.05; ^**^ *p* < 0.01; ^***^ *p* < 0.001.

To explore the proportion of TILs in breast cancer tissues, we utilized the CIBERSORT algorithm to stratify each immune cell subset from TCGA-BRCA datasets and GSE114082 datasets. The results showed that in normal and tumor samples, the TILs with significantly statistical differences included M2 macrophages, M0 macrophages, resting memory CD4 T cells, naïve B cells, and resting mast cells between breast normal tissues and breast cancer tissues (**Figure 2A** and **Supplementary Figures 2A, B**). Additionally, the proportion of TILs with significantly statistical differences in sample with targeted molecular therapy included M0 macrophages, M2 macrophages, resting memory CD4 T cells, naive B cells, and M1 macrophages (**Figure 2B** and **Supplementary Figure 2C**).

Based on the GSE114082 datasets, we identified that the TILs with significant differences were M2 macrophages, M0 macrophages, gamma delta T cells, plasma cells, resting memory CD4 T cells, and resting mast cells in breast cancer tissues pre- or posttreated by trastuzumab. Of these cells, M2 macrophages, gamma delta T cells, resting mast cells, and M0 macrophages were significantly higher in the posttreated breast cancer tissues than those of the pretreated cancer tissues (**Figure 2C** and **Supplementary Figures 2D, E**). The results suggested that tumor tissues own a specific proportion profile of TILs and administration of trastuzumab for breast cancers can rebuild the profile of TILs in the tumor microenvironment of breast cancer.

### Identification of DEGs and Immune-Related Genes

**Figure 3:**
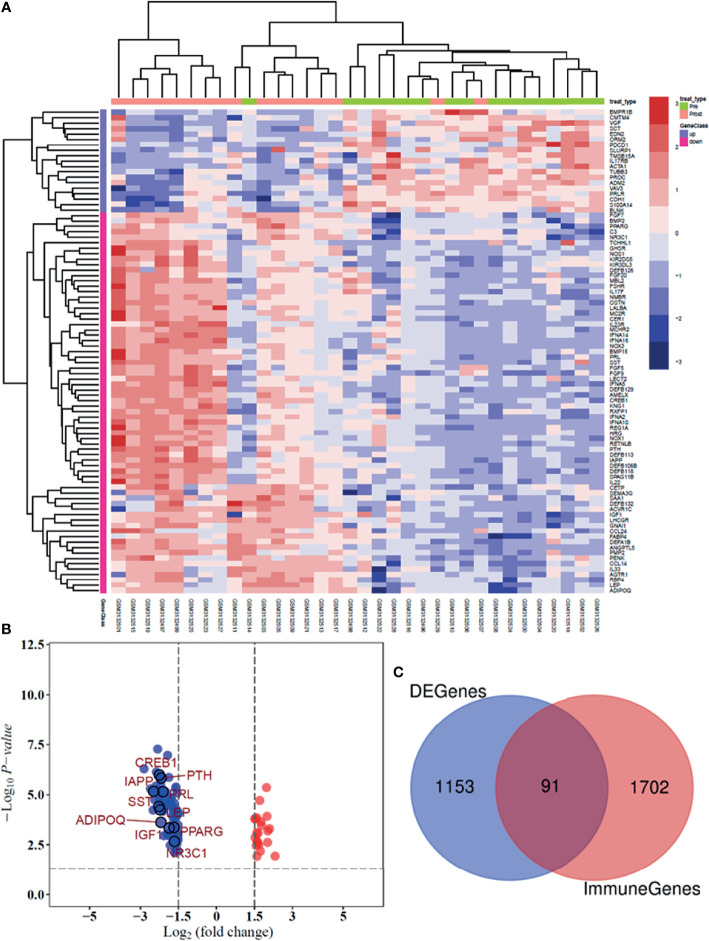
Heatmap and volcano plot presenting the significant immune-related DEGs between patients pre- and posttreated by trastuzumab. **(A)** Heatmap shows the differential expression of 91 DEGs in breast tumor tissues. **(B)** The volcano plot was illustrated the significant difference between genes and 10 hub genes. **(C)** Venn plot was used to identify the common immune-related genes between GSE 114082 dataset and the ImmPort database.

In the GSE114082 dataset, 1,244 DEGs were identified based on an adjusted *p* value of <0.05 and absolute log2FC of >1.5 or <−1.5. Of these genes, 250 were upregulated and 994 were downregulated. We utilized heatmap and volcano plots to visualize the top 91 common immune-related DEGs and 10 hub genes (**Figures 3A, B** **)**. The consistently upregulated and downregulated genes between DEGs and immune-related genes were identified *via* Venn analysis, and a Venn diagram was used to depict the 91 common immune-related DEGs (**Figure 3C**).

### Function Enrichment Analysis

**Figure 4:**
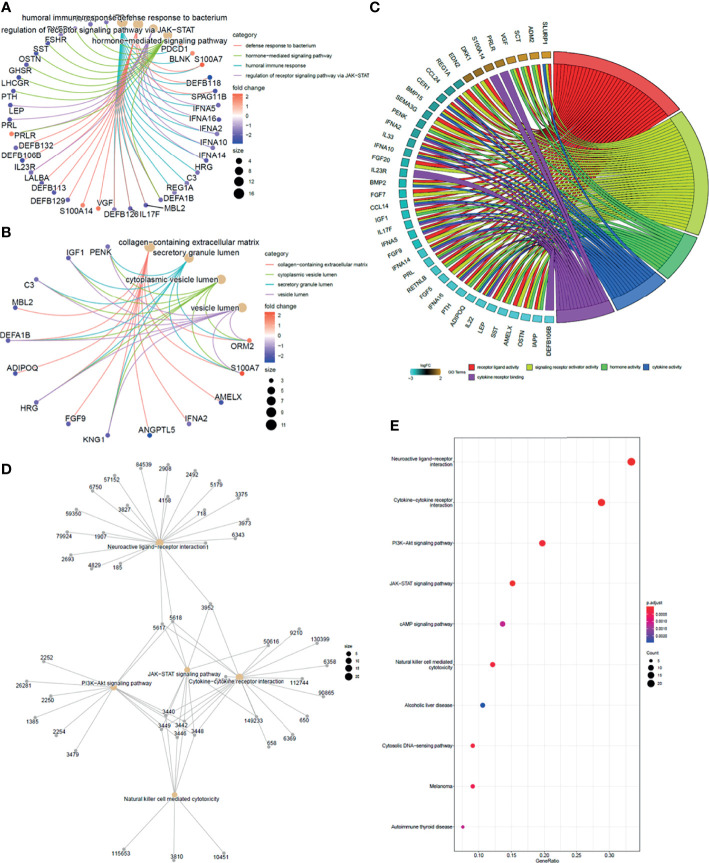
Function enrichment analysis of overlapping DEGs. **(A)** GO cluster plot showing a chord profile circle of biological processes (BP) of the expression spectrum. **(B)** GO cluster plot showing a chord profile circle of cellular components (CC). **(C)** GO cluster plot showing a chord dendrogram of molecular functions (MF) of the expression spectrum. **(D)** KEGG pathway enrichment network. The nodes represent the significantly enriched genes on the specific signaling pathway. **(E)** The *y*-axis is the KEGG pathway enriched terms, and the *x*-axis is the fold of enrichment. The size of the dot means the abundance of enriched genes under the term.

To take insights into the biological functions of the common DEGs in trastuzumab treatment of breast cancer, a total of 91 common immune-related DEGs were utilized for GO enrichment analysis and KEGG pathway enrichment analysis. In the BP term, the results illustrated that the common DEGs were mainly enriched in defense response to the bacterium, humoral immune response, regulation of receptor signaling pathway *via* Janus kinase-signal transducer of activation of transcription (JAK-STAT), hormone-mediated signaling pathway, and antimicrobial humoral immune response mediated by antimicrobial peptide (**Figure 4A**). In the cellular component (CC) term, the common DEGs were mainly associated with secretory granule lumen, cytoplasmic vesicle lumen, platelet alpha granule lumen, vesicle lumen, and collagen-containing extracellular matrix (**Figure 4B**). In the molecular function (MF) term, the common DEGs were mainly involved in the signaling receptor activator activity, receptor-ligand activity, cytokine activity, hormone activity, and cytokine receptor binding (**Figure 4C**).

Enrichment analysis for the 91 unique immune genes identified 17 related KEGG pathways (*p* < 0.01). Meanwhile, the common immune-related DEGs were mainly involved in the cytokine–cytokine receptor interaction, neuroactive ligand–receptor interaction, JAK-STAT signaling pathway, PI3K-Akt signaling pathway, and natural killer cell-mediated cytotoxicity (**Figures 4D, E** **)** (**Supplementary Table 2**).

### PPI Network and Identification of Hub Genes

**Figure 5:**
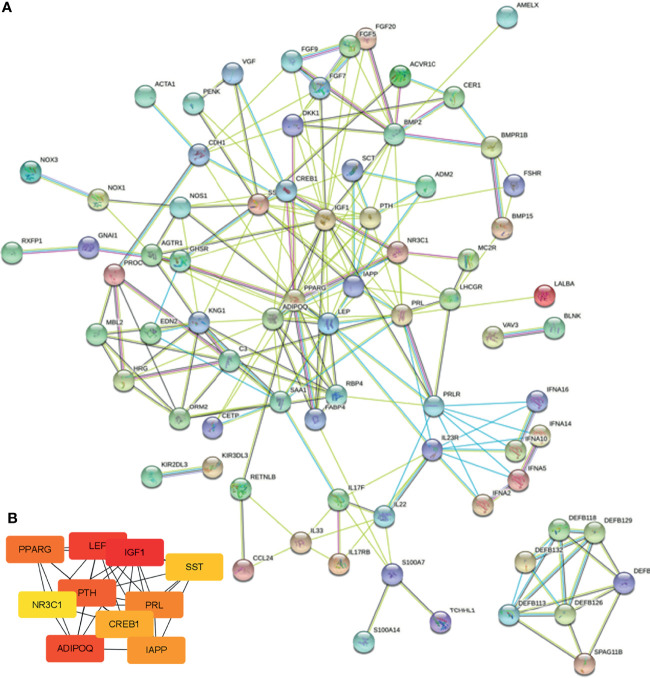
PPI network and hub gene identification. **(A)** PPI network was constructed by all the 91 common immune-related DEGs using the STRING database. **(B)** The top 10 hub genes in the PPI network were screened by Cytoscape plugin cytoHubba.

To further explore the interaction between the common immune-related DEGs and identify the hub genes involved in this process, we conducted a PPI network using the STRING database and Cytoscape. As shown in **Figure 5A**, there were 91 nodes and 214 edges. The top 10 hub genes in the PPI network were screened by cytoHubba, and the top 10 hub genes were insulin-like growth factor-1 (IGF1), lipopolysaccharide elimination protein (LEP), parathyroid hormone (PTH), adiponectin (ADIPOQ), peroxisome proliferator-activated receptor gamma (PPARG), prolactin (PRL), islet amyloid polypeptide (IAPP), cAMP response element-binding protein 1 (CREB1), somatostatin (SST), and nuclear receptor subfamily 3 group C member 1 (NR3C1) (**Figure 5B**).

### Survival Prognostic Model Construction

**Figure 6:**
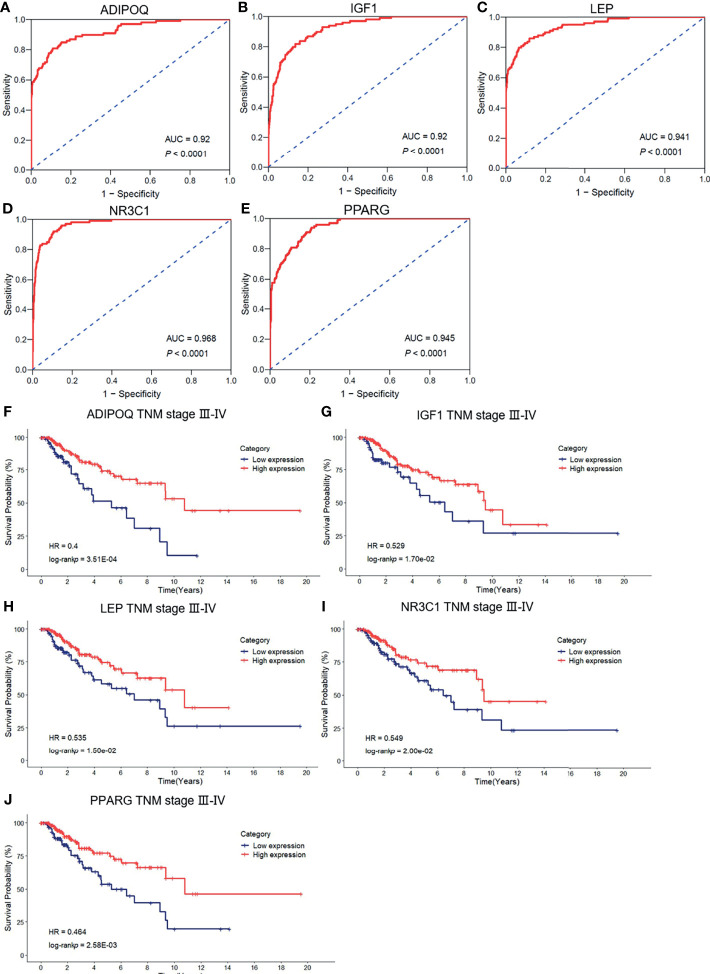
The ROC curve of the risk score in nontumor tissues and breast cancer tissues. **(A–E)** The ROC curve of the validation of the diagnostic value of five immune-related hug genes. **(F–J)** Kaplan–Meier survival analysis for the subgroup of overall survival (OS) based on tumor–node–metastasis (TNM) stages.

To evaluate the diagnostic values of the top 10 hub genes for breast cancer patients, we employed the ROC curves based on the TCGA-BRCA database to assess the diagnostic efficiency. As shown in **Figures 6A–E**, the area under the curves (AUC) of IGF1, LEP, ADIPOQ, CREB1 NR3C1, PPARG, and PRL were 0.92, 0.941, 0.92, 0.721, 0.968, 0.945, and 0.613, respectively. Subsequently, a Kaplan–Meier plotter was employed to assess the prognostic value of the ten hub gens. Based on the TCGA-BRCA dataset, our results showed that the low expression levels of ADIPOQ (HR = 0.4, *p* < 0.001), IGF1 (HR = 0.529, *p* < 0.05), LEP (HR = 0.535, *p* < 0.05), NR3C1 (HR = 0.549, *p* < 0.05), and PPARG (HR = 0.464, *p* < 0.01) were significantly associated with the worse OS of breast cancer patients with tumor pathological stages III–IV (**Figures 6F–J**). Furthermore, the low expression levels of ADIPOQ (HR = 0.416, *p* < 0.01), IGF1 (HR = 0.458, *p* < 0.05), LEP (HR = 0.527, *p* < 0.05), NR3C1 (HR = 0.457, *p* < 0.05), and PPARG (HR = 0.491, *p* < 0.05) were notably related to the poorer BCSS of breast cancer patients with tumor pathological stages III–IV. The low expression levels of NR3C1 (HR = 0.397, *p* < 0.05) were remarkably associated with the poorer BCSS of patients with tumor pathological stages I–II (**Supplementary Figures 3A–F**).

To identify the association between the expression levels of these five hub genes and metastasis risk in breast cancer, we used survival information of breast cancer patients to perform survival analysis for the five hub genes. Of the data collected, low expression levels of three hub genes, ADIPOQ (HR = 0.142, *p* < 0.001), LEP (HR = 0.194, *p* < 0.01), and PPARG (HR = 0.278, *p* < 0.05), were significantly associated with worse DMFS (**Supplementary Figures 3G–I**). Meanwhile, we determined the significant differential expression of the five hub genes between tumor tissues and nontumor tissues (**Supplementary Figure 4**). Overall, these findings confirmed the prognostic value and the relationships between the four hub genes and the metastasis of breast cancer.

## Discussion

Malignant solid tumor tissues, including breast cancer, were composed not only of tumor cells but also normal stromal cells, immune cells, and vascular cells. Accumulating evidence supported the notion that tumor stromal cells play a pivotal role in tumor initiation and metastasis (17). Cancer-associated fibroblasts (CAFs), for example, are consisted of the majority of tumor stroma, especially in breast cancer, and are thought to be novel potential therapeutic targets of estimation in several clinical trials (18–20). The prognostic values of TILs have been estimated in several studies that persistently showed that among patients with triple-negative breast cancer, TILs were related to an improvement of clinical outcome (21). Moreover, a prospective-retrospective study of phase 3 clinical trial revealed that TILs were notably prognostic in the estrogen receptor (ER)-negative/HER2-negative subtypes, which are related to higher disease-free survival and OS (22). However, this phenomenon failed to be observed in HER2-positive subgroups (22). Moreover, a prospective-retrospective analysis of the herceptin in TNBC patients indicated that with the increase of TILs, the relative risk of distant recurrence was reduced (13).

Here, we take a new algorithm, ESTIMATE that utilizes the properties of the transcriptional profiles of cancer samples to demonstrate the tumor cellularity and the different infiltrating normal cells (15), to assess the stromal and immune cells in breast tumor tissues. A significant reduction in stromal signature scores was observed in tumor samples compared with the normal samples, as well as in tumor tissues pretreated by trastuzumab than in tumor tissues posttreated. However, administration of trastuzumab in patients with breast cancer remarkably upregulated the stromal scores in comparison with pretreated trastuzumab. Moreover, lower immune scores were observed in normal samples and pretreated tumor samples, but there were no significant statistical differences. However, the negative or positive immune scores in breast tumor tissues were estimated as independent risk factors for the overall survival of breast cancer patients.

TILs were triggered at an early stage of tumors and can recognize tumor cells to generate large amounts of cytokines (such as IFN-γ) or directly transformed cells (such as natural killer cells and cytotoxic T lymphocyte (CTL) cells), causing the elimination of tumor cells (23). In this study, we utilized the CIBERSORT algorithm that evaluates a large number of tumor samples characterized by RNA sequencing, to identify the proportion of 22 lymphocytes in breast cancer tissues. Our result profiled the percentages and majority of each lymphocyte in the breast tumor tissues whether it was treated by trastuzumab or not. The attendance of pivotal cell populations of the innate and adaptive immune system has been illustrated in breast cancer (24, 25). For instance, tumor-associated macrophages (TAMs) are usually stratified into two main categories, namely classically activated M1 and alternatively activated M2 macrophages, according to the major biological processes in the pathogenesis of tumors (26).

Of these TAMs, the M1 phenotype is thought to be involved in a proinflammatory microenvironment and antitumor immune reactions by the production of type I cytokines (such as IL-12, TNF-α, and nitric oxide synthase). In contrast, the M2 phenotype is related to the immunosuppression in the tumor microenvironment by generating cytokines such as IL-4 and IL-13 and establishing a permissive environment for tumor progression (27). However, our results demonstrated that the M1 phenotype presented higher levels in nontumor tissues than tumor tissues, and this tendency was reversed by the administration of trastuzumab. Meanwhile, a significant reduction of M2 phenotype was observed in tumor tissues than nontumor tissues and reversed by treatment of trastuzumab. This phenomenon may be explained by the phenotype transition of TAMs that is mediated by its interaction with breast cancer cells, showing tumor subtype dependence. For instance, the coculture of TAMs with estrogen receptor-positive breast cancer cells stimulates the generation of M1 phenotype, while the coculture of TAMs with triple-negative breast cells enhances the proliferation of M2 phenotype (28, 29). In our study, macrophages still consisted of the main proportion of immune-infiltrating cells in breast tumor tissues, as well as in tumor tissues treated by trastuzumab.

In this study, we focused on the genetic changes in transcription level between pre- and posttreatment of trastuzumab in breast cancer patients to investigate the effect of trastuzumab on the expression of immune-related genes. GO and KEGG enrichment analyses revealed that 91 DEGs mainly enriched in humoral immune response, regulation of receptor signaling pathway *via* JAK-STAT, antimicrobial humoral immune response mediated by an antimicrobial peptide, signaling receptor activator activity, cytokine activity, PI3K-Akt signaling pathway, and natural killer cell-mediated cytotoxicity. Of them, the JAK-STAT signaling pathway involves almost all immune regulatory processes, including tumor cell recognition, tumor-driven immune evasion, and antitumor immune responses (30). Therefore, this pathway has become a potently attractive therapeutic target, and relatively therapeutic strategies (such as tofacitinib targeting JAK3, ruxolitinib as an inhibitor of JAK1 and JAK2, oclacitinib as a pan-JAK inhibitor) obtained positive effects in preclinical and clinical models (31–34). The PI3K/Akt signaling pathway is involved in the essential cellular biological process, such as cell proliferation, apoptosis, and angiogenesis (35).

Hub genes were identified based on the protein–protein interaction (PPI) network, and then the 10 hub genes were verified using TCGA-BRCA datasets. ROC curves revealed that 7 hub genes presented a credible classification effect between tumor and normal tissues and may be involved in the response of trastuzumab in breast cancer patients. To further explore the prognostic values of hub genes, a survival analysis was performed based on TCGA-BRCA datasets. Lower expression levels of five hub gens, including IGF1, LEP, ADIPOQ, NR3C1, and PPARG, were determined and were remarkably associated with poorer OS and BCSS among breast cancer patients. Additionally, of these hub genes, three (ADIPOQ, LEP, and PPARG) were significantly related to the poorer DMFS. Therefore, we considered IGF1, LEP, ADIPOQ, NR3C1, and PPARG as potential predictors for the poor prognosis of patients with breast cancer. The IGF1 is a polypeptide that is mediated mitogenic and anti-apoptotic effects via IGF1 receptor that is a type 2 tyrosine kinase receptor, and the accumulating evidence indicated that IGF axis played pivotal roles in human cancer progression and can be targets for therapeutic intervention (36, 37).

Although this study provided a viewpoint on the use of the immune score in the prognosis of breast cancer, it still has some shortcomings. For instance, the study depended on retrospective data when prospective data are needed to verify these outcomes. In conclusion, this study explored the composition of stroma and immune infiltration cells and profiled the percentage of tumor-related infiltrating lymphocytes in the tumor microenvironment of breast tumor tissues whether treated by trastuzumab or not. Understanding the tumor immune microenvironment provides a pivotal insight that would improve the diagnosis and response of targeted molecular therapy. Meanwhile, a novel five gene-based immune gene signature from breast cancer could predict prognostic factors of the poor overall survival for patients with breast cancer treated with trastuzumab.

## Data Availability Statement

The datasets presented in this study can be found in online repositories. The names of the repository/repositories and accession number(s) can be found in the article/**Supplementary Material**.

## Author Contributions

FC contributed to the study concept, design, analysis of data, and the drafting of the manuscript. JF contributed to the study concept and manuscript reviews. All authors listed have made a substantial, direct, and intellectual contribution to the work and approved it for publication.

## Conflict of Interest

Author FC was employed by RemeGen Co. Ltd.

The remaining author declares that the research was conducted in the absence of any commercial or financial relationships that could be construed as a potential conflict of interest.

## Publisher’s Note

All claims expressed in this article are solely those of the authors and do not necessarily represent those of their affiliated organizations, or those of the publisher, the editors and the reviewers. Any product that may be evaluated in this article, or claim that may be made by its manufacturer, is not guaranteed or endorsed by the publisher.

## References

[1] Wu T Dai Y. Tumor Microenvironment and Therapeutic Response. Cancer Lett (2017) 387:61–8. doi: 10.1016/j.canlet.2016.01.043 26845449

[2] Meads MB Gatenby RA Dalton WS. Environment-Mediated Drug Resistance: A Major Contributor to Minimal Residual Disease. Nat Rev Cancer (2009) 9(9):665–74. doi: 10.1038/nrc2714 19693095

[3] Maibach F Sadozai H Seyed Jafari SM Hunger RE Schenk M. Tumor-Infiltrating Lymphocytes and Their Prognostic Value in Cutaneous Melanoma. Front Immunol (2020) 11:2105. doi: 10.3389/fimmu.2020.02105 33013886 PMC7511547

[4] Sato E Olson SH Ahn J Bundy B Nishikawa H Qian F. Intraepithelial CD8+ Tumor-Infiltrating Lymphocytes and a High CD8+/regulatory T Cell Ratio are Associated With Favorable Prognosis in Ovarian Cancer. Proc Natl Acad Sci USA (2005) 102(51):18538–43. doi: 10.1073/pnas.0509182102 PMC1311741 16344461

[5] Diederichsen AC Hjelmborg J Christensen PB Zeuthen J Fenger C. Prognostic Value of the CD4+/CD8+ Ratio of Tumour Infiltrating Lymphocytes in Colorectal Cancer and HLA-DR Expression on Tumour Cells. Cancer Immunol Immunother: CII (2003) 52(7):423–8. doi: 10.1007/s00262-003-0388-5 PMC11032970 12695859

[6] Burugu S Asleh-Aburaya K Nielsen TO. Immune Infiltrates in the Breast Cancer Microenvironment: Detection, Characterization and Clinical Implication. Breast Cancer (Tokyo Japan) (2017) 24(1):3–15. doi: 10.1007/s12282-016-0698-z 27138387

[7] Park YH Lal S Lee JE Choi YL Wen J Ram S. Chemotherapy Induces Dynamic Immune Responses in Breast Cancers That Impact Treatment Outcome. Nat Commun (2020) 11(1):6175. doi: 10.1038/s41467-020-19933-0 33268821 PMC7710739

[8] Stanton SE Disis ML. Clinical Significance of Tumor-Infiltrating Lymphocytes in Breast Cancer. J Immunother Cancer (2016) 4:59. doi: 10.1186/s40425-016-0165-6 27777769 PMC5067916

[9] Varn FS Mullins DW Arias-Pulido H Fiering S Cheng C. Adaptive Immunity Programmes in Breast Cancer. Immunology (2017) 150(1):25–34. doi: 10.1111/imm.12664 27564847 PMC5341497

[10] Emens LA Ascierto PA Darcy PK Demaria S Eggermont AMM Redmond WL. Cancer Immunotherapy: Opportunities and Challenges in the Rapidly Evolving Clinical Landscape. Eur J Cancer (Oxford England: 1990) (2017) 81:116–29. doi: 10.1016/j.ejca.2017.01.035 28623775

[11] Lipson EJ Forde PM Hammers HJ Emens LA Taube JM Topalian SL. Antagonists of PD-1 and PD-L1 in Cancer Treatment. Semin Oncol (2015) 42(4):587–600. doi: 10.1053/j.seminoncol.2015.05.013 26320063 PMC4555873

[12] Tumeh PC Harview CL Yearley JH Shintaku IP Taylor EJ Robert L. PD-1 Blockade Induces Responses by Inhibiting Adaptive Immune Resistance. Nature (2014) 515(7528):568–71. doi: 10.1038/nature13954 PMC4246418 25428505

[13] Loi S Michiels S Salgado R Sirtaine N Jose V Fumagalli D. Tumor Infiltrating Lymphocytes are Prognostic in Triple Negative Breast Cancer and Predictive for Trastuzumab Benefit in Early Breast Cancer: Results From the FinHER Trial. Ann Oncol: Off J Eur Soc Med Oncol (2014) 25(8):1544–50. doi: 10.1093/annonc/mdu112 24608200

[14] Adamczyk A Kruczak A Harazin-Lechowska A Ambicka A Grela-Wojewoda A Domagała-Haduch M. Relationship Between HER2 Gene Status and Selected Potential Biological Features Related to Trastuzumab Resistance and Its Influence on Survival of Breast Cancer Patients Undergoing Trastuzumab Adjuvant Treatment. OncoTargets Ther (2018) 11:4525–35. doi: 10.2147/OTT.S166983 PMC6082350 30122944

[15] Yoshihara K Shahmoradgoli M Martínez E Vegesna R Kim H Torres-Garcia W. Inferring Tumour Purity and Stromal and Immune Cell Admixture From Expression Data. Nat Commun (2013) 4:2612. doi: 10.1038/ncomms3612 24113773 PMC3826632

[16] Newman AM Liu CL Green MR Gentles AJ Feng W Xu Y. Robust Enumeration of Cell Subsets From Tissue Expression Profiles. Nat Methods (2015) 12(5):453–7. doi: 10.1038/nmeth.3337 PMC4739640 25822800

[17] Mao Y Keller ET Garfield DH Shen K Wang J. Stromal Cells in Tumor Microenvironment and Breast Cancer. Cancer Metastasis Rev (2013) 32(1-2):303–15. doi: 10.1007/s10555-012-9415-3 PMC4432936 23114846

[18] Aprelikova O Palla J Hibler B Yu X Greer YE Yi M. Silencing of miR-148a in Cancer-Associated Fibroblasts Results in WNT10B-Mediated Stimulation of Tumor Cell Motility. Oncogene (2013) 32(27):3246–53. doi: 10.1038/onc.2012.351 PMC3711253 22890324

[19] Devarajan E Song YH Krishnappa S Alt E. Epithelial-Mesenchymal Transition in Breast Cancer Lines is Mediated Through PDGF-D Released by Tissue-Resident Stem Cells. Int J Cancer (2012) 131(5):1023–31. doi: 10.1002/ijc.26493 22038895

[20] Quail DF Joyce JA. Microenvironmental Regulation of Tumor Progression and Metastasis. Nat Med (2013) 19(11):1423–37. doi: 10.1038/nm.3394 PMC3954707 24202395

[21] Adams S Gray RJ Demaria S Goldstein L Perez EA Shulman LN. Prognostic Value of Tumor-Infiltrating Lymphocytes in Triple-Negative Breast Cancers From Two Phase III Randomized Adjuvant Breast Cancer Trials: ECOG 2197 and ECOG 1199. J Clin Oncol: Off J Am Soc Clin Oncol (2014) 32(27):2959–66. doi: 10.1200/JCO.2013.55.0491 PMC4162494 25071121

[22] Loi S Sirtaine N Piette F Salgado R Viale G Van Eenoo F. Prognostic and Predictive Value of Tumor-Infiltrating Lymphocytes in a Phase III Randomized Adjuvant Breast Cancer Trial in Node-Positive Breast Cancer Comparing the Addition of Docetaxel to Doxorubicin With Doxorubicin-Based Chemotherapy: BIG 02-98. J Clin Oncol: Off J Am Soc Clin Oncol (2013) 31(7):860–7. doi: 10.1200/JCO.2011.41.0902 23341518

[23] Shankaran V Ikeda H Bruce AT White JM Swanson PE Old LJ. IFNgamma and Lymphocytes Prevent Primary Tumour Development and Shape Tumour Immunogenicity. Nature (2001) 410(6832):1107–11. doi: 10.1038/35074122 11323675

[24] Tang X. Tumor-Associated Macrophages as Potential Diagnostic and Prognostic Biomarkers in Breast Cancer. Cancer Lett (2013) 332(1):3–10. doi: 10.1016/j.canlet.2013.01.024 23348699

[25] Wagner J Rapsomaniki MA Chevrier S Anzeneder T Langwieder C Dykgers A. A Single-Cell Atlas of the Tumor and Immune Ecosystem of Human Breast Cancer. Cell (2019) 177(5):1330–45.e18. doi: 10.1016/j.cell.2019.03.005 30982598 PMC6526772

[26] Qiu SQ Waaijer SJH Zwager MC de Vries EGE van der Vegt B Schröder CP. Tumor-Associated Macrophages in Breast Cancer: Innocent Bystander or Important Player? Cancer Treat Rev (2018) 70:178–89. doi: 10.1016/j.ctrv.2018.08.010 30227299

[27] Qian BZ Pollard JW. Macrophage Diversity Enhances Tumor Progression and Metastasis. Cell (2010) 141(1):39–51. doi: 10.1016/j.cell.2010.03.014 20371344 PMC4994190

[28] Hollmén M Roudnicky F Karaman S Detmar M. Characterization of Macrophage–Cancer Cell Crosstalk in Estrogen Receptor Positive and Triple-Negative Breast Cancer. Sci Rep (2015) 5:9188. doi: 10.1038/srep09188 25776849 PMC4361875

[29] Vallerand D Massonnet G Kébir F Gentien D Maciorowski Z de la Grange P. Characterization of Breast Cancer Preclinical Models Reveals a Specific Pattern of Macrophage Polarization. PloS One (2016) 11(7):e0157670. doi: 10.1371/journal.pone.0157670 27388901 PMC4936680

[30] Owen KL Brockwell NK Parker BS. JAK-STAT Signaling: A Double-Edged Sword of Immune Regulation and Cancer Progression. Cancers (2019) 11(12):1–26. doi: 10.3390/cancers11122002 PMC6966445 31842362

[31] Kudlacz E Perry B Sawyer P Conklyn M McCurdy S Brissette W. The Novel JAK-3 Inhibitor CP-690550 is a Potent Immunosuppressive Agent in Various Murine Models. Am J Transplant: Off J Am Soc Transplant Am Soc Transplant Surgeons (2004) 4(1):51–7. doi: 10.1046/j.1600-6143.2003.00281.x 14678034

[32] Kremer JM Cohen S Wilkinson BE Connell CA French JL Gomez-Reino J. A Phase IIb Dose-Ranging Study of the Oral JAK Inhibitor Tofacitinib (CP-690,550) Versus Placebo in Combination With Background Methotrexate in Patients With Active Rheumatoid Arthritis and an Inadequate Response to Methotrexate Alone. Arthritis Rheum (2012) 64(4):970–81. doi: 10.1002/art.33419 22006202

[33] Clark JD Flanagan ME Telliez JB. Discovery and Development of Janus Kinase (JAK) Inhibitors for Inflammatory Diseases. J Medicinal Chem (2014) 57(12):5023–38. doi: 10.1021/jm401490p 24417533

[34] Cosgrove SB Wren JA Cleaver DM Walsh KF Follis SI King VI. A Blinded, Randomized, Placebo-Controlled Trial of the Efficacy and Safety of the Janus Kinase Inhibitor Oclacitinib (Apoquel^®^) in Client-Owned Dogs With Atopic Dermatitis. Vet Dermatol (2013) 24(6):587–97, e141-2. doi: 10.1111/vde.12088 24581322 PMC4286885

[35] Miricescu D Totan A Stanescu S II Badoiu SC Stefani C Greabu M. PI3K/AKT/mTOR Signaling Pathway in Breast Cancer: From Molecular Landscape to Clinical Aspects. Int J Mol Sci (2020) 22(1):173–97. doi: 10.3390/ijms22010173 PMC7796017 33375317

[36] Samani AA Yakar S LeRoith D Brodt P. The Role of the IGF System in Cancer Growth and Metastasis: Overview and Recent Insights. Endocrine Rev (2007) 28(1):20–47. doi: 10.1210/er.2006-0001 16931767

[37] Murphy N Knuppel A Papadimitriou N Martin RM Tsilidis KK Smith-Byrne K. Insulin-Like Growth Factor-1, Insulin-Like Growth Factor-Binding Protein-3, and Breast Cancer Risk: Observational and Mendelian Randomization Analyses With ∼430 000 Women. Ann Oncol: Off J Eur Soc Med Oncol (2020) 31(5):641–9. doi: 10.1016/j.annonc.2020.01.066 PMC7221341 32169310

