# Supplementary material for: Benefits of Targeted Molecular Therapy to Immune Infiltration and Immune-Related Genes Predicting Signature in Breast Cancer
Source: Front Oncol. 2022 Mar 4;12:824166. doi: 10.3389/fonc.2022.824166 (PMC8934425; doi:10.3389/fonc.2022.824166)

**Supplementary Figure 1.** The quantality assessment of the GEO dataset. GSE114082 dataset was assessed by box plot.


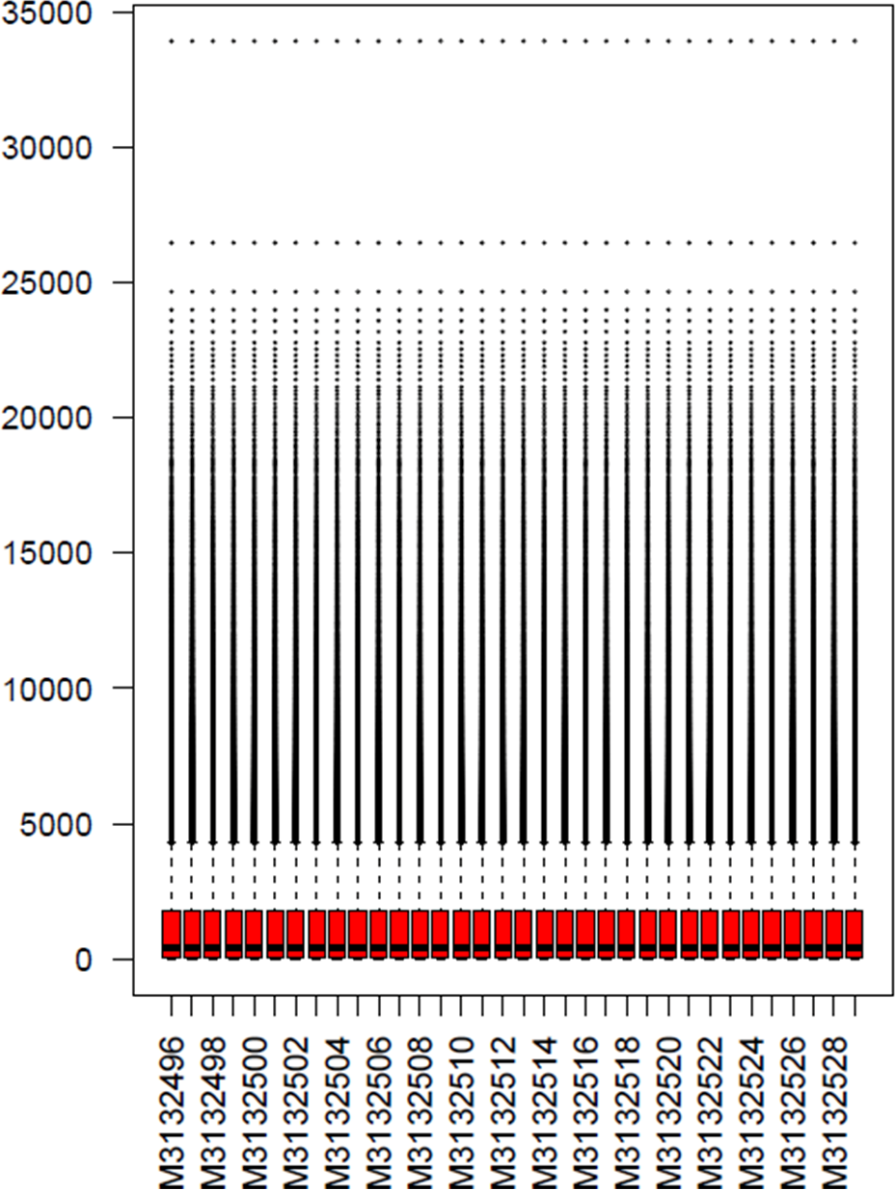


**Supplementary Figure 2**. The proportion of tumor-related infiltrating lymphocytes in breast tumor tissues. (A) The percentages of tumor-related infiltration lymphocytes in non-tumor tissues. (B) The percentages of tumor-related infiltration lymphocytes in breast tumor tissues. (C) The percentages of tumor-related infiltration lymphocytes in breast tumor tissues with treated by targeted molecular therapy. (D) The percentages of tumor-related infiltration lymphocytes in breast tumor tissues before treated by trastuzumab. (E) The percentages of tumor-related infiltration lymphocytes in breast tumor tissues after treated by trastuzumab.


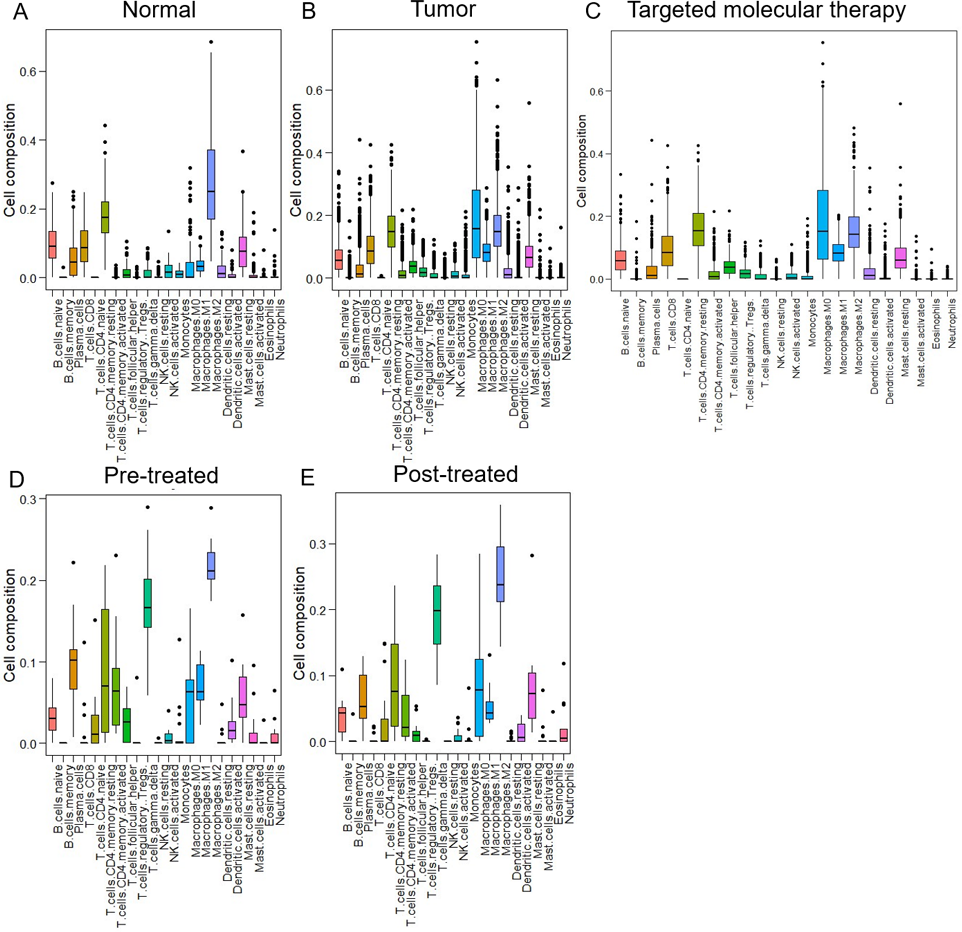


**Supplementary Figure 3.** Breast cancer specific survival (BCSS) and distant metastasis free survival (DMFS) were estimated using the Kaplan-Meier method and compared using the log-rang test. (A-F) The five hub genes were estimated on BCSS. (G-I) The three hug genes were investigated on DMFS.


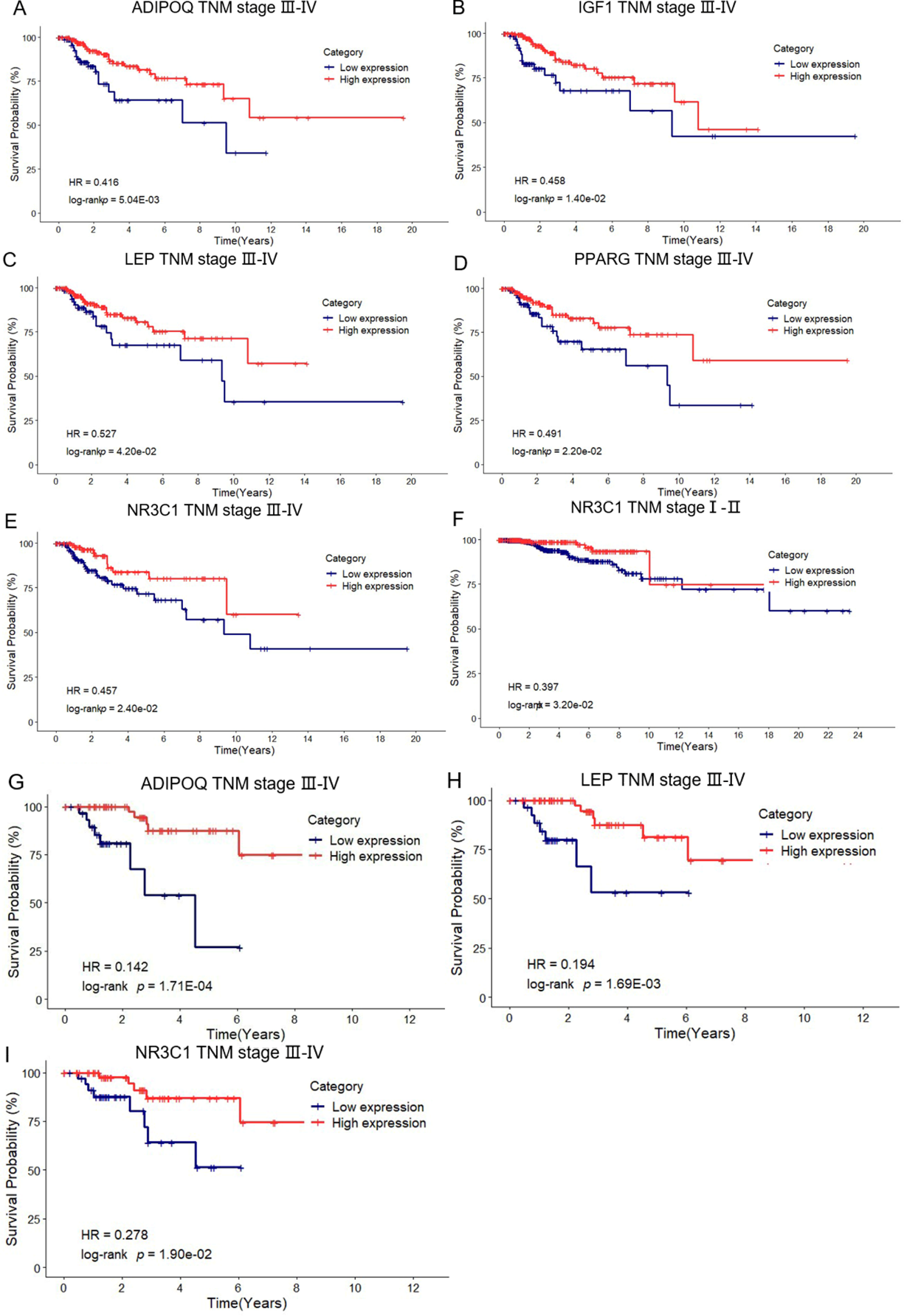


**Supplementary Figure 4**. Comparison of the five hub genes mRNA expression in breast cancer tissues and non-tumor tissues.


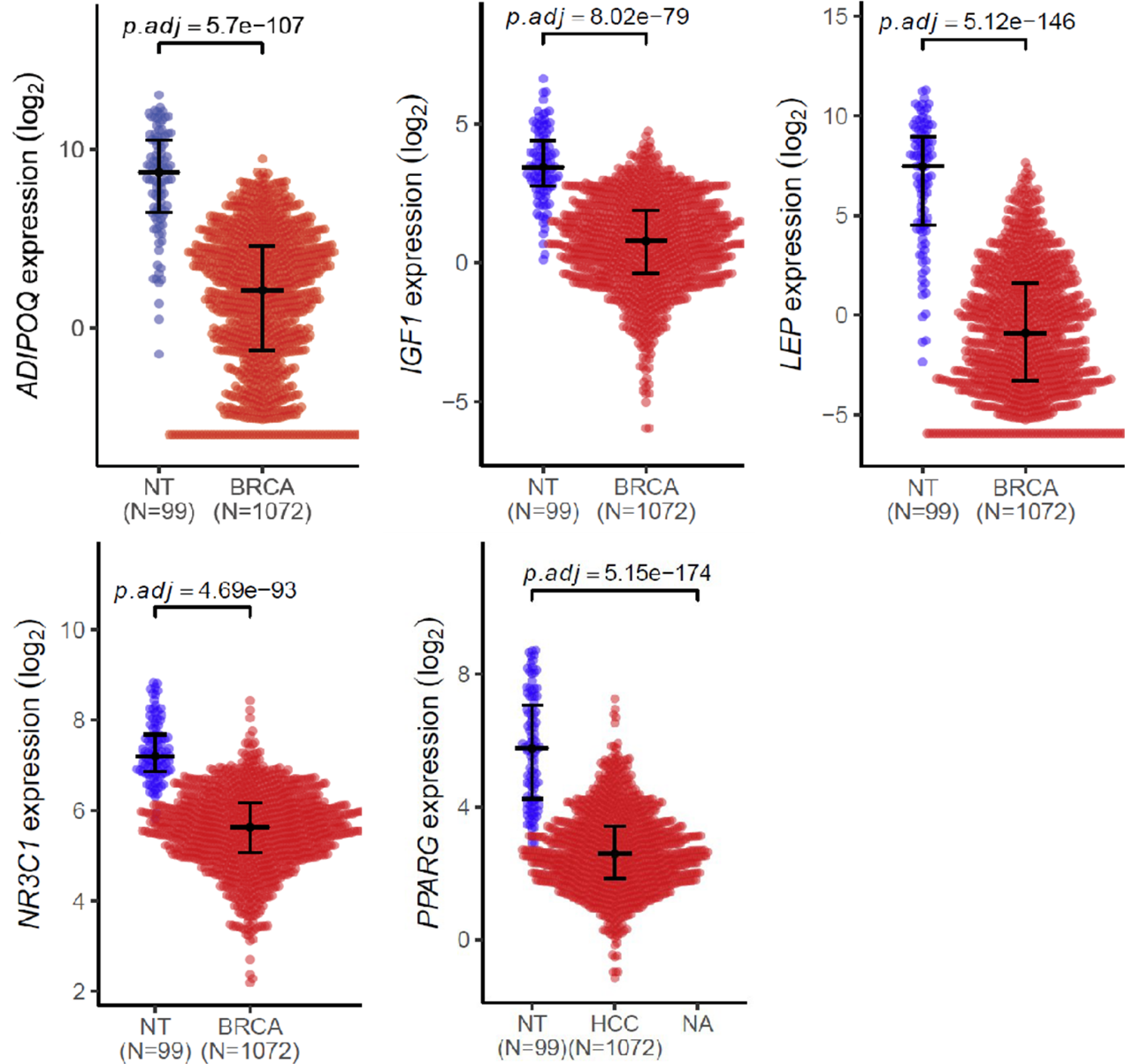

Supplement: Supplementary file 1 [file DataSheet_1.docx]
