# Supplementary material for: Benefits of Targeted Molecular Therapy to Immune Infiltration and Immune-Related Genes Predicting Signature in Breast Cancer
Source: Front Oncol. 2022 Mar 4;12:824166. doi: 10.3389/fonc.2022.824166 (PMC8934425; doi:10.3389/fonc.2022.824166)
Supplement: Supplementary file 2 [file Table_1.docx]

**Supplementary Table 1**. Cox proportional hazards regression model analysis of overall survival according to targeted molecular therapy.

| Variables | Univariate analysis | | Multivariate analysis | |
| --- | --- | --- | --- | --- |
|  | HR (95% CI) | ***P*** | HR (95% CI) | ***P*** |
| Age (≥ 65 vs. <65) | 2.17(1.56,3.03) | 0.056 | - | - |
| TNM stage (Ⅱ vs. Ⅰ) | 1.71(0.98,3) | 0.163 | 2.84(0.66,12.3) | 0.163 |
| TNM stage (Ⅲ vs. Ⅰ) | 3.17(1.76,5.71) | **0.048** | 4.64(1.01,21.12) | **0.048** |
| TNM stage (Ⅳ vs. Ⅰ) | 13.48(6.57,27.66) | **<0.001** | 33.99(6.5,177.81) | **<0.001** |
| StromalScore (positive vs. negative) | 1.08(0.76,1.53) | 0.599 | - | - |
| ImmuneScore (positive vs. negative) | 0.63(0.45,0.87) | 0.088 | - | - |

Statistically significant p values are given in bold, *P* < 0.05

HR hazard ratio, CI confidence interval

**Supplementary** **Table 2**. KEGG pathway enrichment analysis.

| Term | P-value | Genes |
| --- | --- | --- |
| Cytokine-cytokine receptor interaction | <0.001 | IFNA5, CCL14, CCL24, IL22, IFNA14, IFNA16, IL23R, IFNA2, PRL, PRLR, IL17RB, BMP2, LEP, BMPR1B, IFNA10 |
| JAK-STAT signaling pathway | <0.001 | IFNA5, IL22, IFNA14, IFNA16, LEP, IL23R, IFNA2, PRL, PRLR, IFNA10 |
| Neuroactive ligand-receptor interaction | <0.001 | GHSR, LHCGR, MC2R, LEP, FSHR, AGTR1, PRL, NMBR, NR3C1, PRLR, RXFP1, MCHR2 |
| PI3K-Akt signaling pathway | <0.001 | IFNA5, IFNA14, IFNA16, IFNA2, PRL, IGF1, PRLR, FGF5, FGF7, CREB1, FGF9, FGF20, IFNA10 |
| Cytosolic DNA-sensing pathway | <0.001 | IFNA5, IL33, IFNA14, IFNA16, IFNA2, IFNA10 |
| Melanoma | <0.001 | FGF5, FGF7, FGF9, CDH1, FGF20, IGF1 |
| Natural killer cell mediated cytotoxicity | <0.001 | IFNA5, VAV3, IFNA14, IFNA16, IFNA2, KIR2DS5, IFNA10 |
| Autoimmune thyroid disease | <0.001 | IFNA5, IFNA14, IFNA16, IFNA2, IFNA10 |
| cAMP signaling pathway | <0.001 | VAV3, GHSR, MC2R, CREB1, FSHR, GNAI1 |
| Tuberculosis | <0.001 | IFNA5, C3, IFNA14, CREB1, IFNA16, IFNA2, IFNA10 |
